# Leptospira Exposure and Waste Pickers: A Case-Control Seroprevalence Study in Durango, Mexico

**DOI:** 10.14740/jocmr2217w

**Published:** 2015-06-09

**Authors:** Cosme Alvarado-Esquivel, Jesus Hernandez-Tinoco, Luis Francisco Sanchez-Anguiano, Agar Ramos-Nevarez, Sandra Margarita Cerrillo-Soto, Carlos Alberto Guido-Arreola

**Affiliations:** a Biomedical Research Laboratory, Faculty of Medicine and Nutrition, Juarez University of Durango State, Durango, Mexico; b Institute for Scientific Research “Dr. Roberto Rivera Damm”, Juarez University of Durango State, Durango, Mexico; c Clinica de Medicina Familiar, Instituto de Seguridad y Servicios Sociales de los Trabajadores del Estado, Predio Canoas S/N, 34079 Durango, Mexico

**Keywords:** *Leptospira*, Epidemiology, Seroprevalence, Waste pickers, Mexico

## Abstract

**Background:**

Infection with *Leptospira* may occur by contact with *Leptospira*-infected animals. Waste pickers are in contact with rodents and dogs while picking in the garbage. Whether waste pickers are at risk for *Leptospira* infection is largely unknown. This study was aimed to determine the association of *Leptospira* IgG seroprevalence with the occupation of waste picking, and to determine the epidemiological characteristics of the waste pickers with *Leptospira* exposure.

**Methods:**

Through a case-control study, we determined the seroprevalence of anti-*Leptospira* IgG antibodies in 90 waste pickers and 90 age- and gender-matched control subjects in Durango City, Mexico using an enzyme immunoassay. Data were analyzed by bivariate and multivariate analyses.

**Results:**

The prevalence of anti-*Leptospira* IgG antibodies was similar in waste pickers (4/90: 4.4%) to that in control subjects (5/90: 5.6%) (P = 1.00). Bivariate analysis showed that *Leptospira* exposure in waste pickers was associated with increasing age (P = 0.009), no education (P = 0.008), and consumption of rat meat (P = 0.04). However, these associations were no longer found by multivariate analysis. *Leptospira* exposure in waste pickers was not associated with health status, duration in the activity, wearing hand gloves and facemasks, history of injuries with sharp material of the garbage, or contact with animals or soil.

**Conclusions:**

This is the first study about *Leptospira* exposure in waste pickers. Results suggest that waste pickers are not at increasing risk for *Leptospira* exposure in Durango City, Mexico. Further research with a larger sample size to elucidate the association of *Leptospira* exposure with waste picking activity is needed.

## Introduction

Leptospirosis is a worldwide zoonotic disease caused by the pathogenic bacteria of the genus *Leptospira* [1, 2]. Transmission of *Leptospira* to humans occurs by direct contact with *Leptospira*-infected animals or indirectly with food, water or soil contaminated with urine from infected animals [3]. Rodents are the most important primary hosts of *Leptospira* [3-5]. However, dogs may play an important role for acquiring *Leptospira* infection due to their proximity to humans [6]. The main route of infection with *Leptospira* is by skin abrasions [3]. Other routes of *Leptospira* infection including mucosa contact, digestive or respiratory are rare or exceptional [3]. The clinical spectrum of *Leptospira* infection varies from asymptomatic to severe disease with multiple organic failure [7-10]. Weil’s disease is a severe form of leptospirosis and it is characterized by fever, jaundice, splenomegaly, nephritis, and hemorrhage [7, 11]. Leptospirosis is often misdiagnosed and under-reported [7].

Waste transfer stations contain a lot of garbage that serve as food for a number of animals. In fact, rats, mice and dogs are readily observed in waste transfer stations. Thus, waste pickers working in waste transfer stations have contact with a considerable number of these animals. This condition may represent a risk for infection with *Leptospira*. Furthermore, skin injuries with sharp materials while waste picking may occur. This condition is an important route of infection with *Leptospira*. To the best of our knowledge, the association of *Leptospira* exposure with waste picking activity has not been studied. Therefore, we sought to determine the association of *Leptospira* IgG antibodies and waste picking activity in Durango City, Mexico and to determine the socio-demographic, work, clinical, and behavioral characteristics of the waste pickers exposed to *Leptospira*.

## Materials and Methods

### Study design and study population

We performed an age- and gender-matched case-control study using residual serum samples from *Toxoplasma gondii* serosurveys in waste pickers [12] and general population [13] in Durango City, Mexico. The group of cases included 90 waste pickers and the group of controls included 90 subjects of the general population without waste picking activity. Cases and controls were compared for the presence of anti-*Leptospira* IgG antibodies. Inclusion criteria for cases were: 1) subjects with waste picking activity in the municipal solid waste transfer station in Durango City, Mexico; 2) aged 14 years and older; 3) waste picking activity for at least 3 months; and 4) who accepted to participate in the study. Gender was not a restrictive criterion for enrollment. Waste pickers enrolled in the study were 14 - 76 (mean: 36.0 ± 17.1) years old, 56 were females and 34 were males. The control group included 56 females and 34 males. Controls had miscellaneous occupations including students, homemakers, employees, and business. The mean age in controls was 35.7 ± 16.8 (range: 18 - 78) years old and similar to that in waste pickers (P = 0.91).

### Socio-demographic, work, clinical, and behavioral data of waste pickers

A questionnaire was submitted to obtain the socio-demographic, work, clinical, and behavioral data of the study population. Age, gender, birthplace, residence, educational level, and socioeconomic status were obtained from all participants. Work items in waste pickers included duration (number of years) in the activity, habitual use of safety practices (use of hand gloves and face masks), history of injuries with sharp material of the garbage, eating or drinking alcohol while waste picking, eating from the garbage, and washing hands before eating. Clinical data included the health status (healthy or ill), and the type of disease if any. Behavioral items included contact with animals, drinking untreated water or unpasteurized milk, consumption of meat and type of meat consumed (pork, lamb, beef, goat, boar, chicken, turkey, rabbit, deer, squirrel, horse, fish, and rat), consumption of unwashed raw vegetables or fruits, and contact with soil (gardening or agriculture). In addition, the housing conditions of the waste pickers were assessed by using the Bronfman’s criteria [14]. Briefly, five variables were examined: number of persons in the house, number of rooms in the house, type of material (soil, ceramic, and wood) of the floor of the house, availability of drinkable water, and form of elimination of excreta.

### Serological detection of *Leptospira* antibodies

Sera from cases and controls were analyzed for detection of anti-*Leptospira* IgG antibodies using a commercially available enzyme-linked immunosorbent assay kit, “*Leptospira* IgG ELISA test” (Diagnostic Automation Inc., Calabasas, CA). A serum sample was considered reactive for anti-*Leptospira* IgG antibodies when an absorbance reading was ≥ 0.5 optical density (OD) units. Absorbance reading between 0.5 and 1.0 OD units was considered weakly reactive. A sample with absorbance reading > 1.0 OD units was considered strongly reactive or with high antibody levels. According to the kit’s insert, this test has a sensitivity of 100% and a specificity of 100%. All assays were performed according to the manufacturer’s instructions. Positive and negative controls were included in each assay.

### Statistical analysis

Results were analyzed with the software Epi Info version 7 and SPSS version 15.0. The Fisher exact test was used to compare frequencies between groups. The association between waste pickers characteristics and *Leptospira* seropositivity was evaluated by bivariate and multivariate analyses. Only variables with a P value ≤ 0.10 obtained in the bivariate analysis were further analyzed by multivariate analysis. Odds ratios (ORs) and 95% confidence intervals (CIs) were calculated by logistic regression analysis with the Enter method. Statistical significance was set at a P value < 0.05.

### Ethical aspects

The Ethical Committee of the Instituto de Seguridad y Servicios Sociales de los Trabajadores del Estado in Durango City approved this project. We examined only residual sera and data of participants from previous surveys [12, 13]. We obtained a written informed consent from all participants.

## Results

The prevalence of anti-*Leptospira* IgG antibodies was similar in waste pickers (4/90: 4.4%) to that in control subjects (5/90: 5.6%) (OR = 0.79; 95% CI: 0.20 - 3.04; P = 1.00). The frequency of high levels (≥ 1.0 OD units) of anti-*Leptospira* IgG antibodies was also similar in waste pickers (1/90: 1.1%) to that in control subjects (1/90: 1.1%) (OR = 1.00; 95% CI: 0.06 - 16.23; P = 1.00).

Of the socio-demographic characteristics of waste pickers, the variables age and educational level were associated with *Leptospira* exposure. Prevalence of *Leptospira* antibodies was significantly (P = 0.008) higher in waste pickers without education than those with education (4/28: 14.3% vs. 0/62: 0%, respectively). Exposure to *Leptospira* increased with age (P = 0.009); seroreactivity was observed only in waste pickers aged 30 years and older. Other socio-demographic characteristics of waste pickers including gender, birthplace, residence, and socioeconomic status showed P values higher than 0.10 by bivariate analysis.

Concerning work characteristics of waste pickers, none of the variables including duration in the activity, use of hand gloves and face masks, history of injuries with sharp material of the garbage, eating or drinking alcohol while waste picking, eating from the garbage, and washing hands before eating showed P values lower than 0.10 by bivariate analysis.

Of the clinical characteristics, the frequency of *Leptospira* exposure was similar in healthy (3/78: 3.8%) to that in ill (1/10: 10%) waste pickers (P = 0.38). The ill waste picker with *Leptospira* antibodies suffered from bronchitis.

With respect to behavioral characteristics of waste pickers, two variables showed P values ≤ 0.10 by bivariate analysis: consumption of rabbit meat and consumption of rat meat. The frequency of *Leptospira* seropositivity was higher in waste pickers with consumption of rabbit meat (3/27: 11.1%) than that in those without this practice (1/63: 1.6%) (P = 0.07). Similarly, the frequency of *Leptospira* seropositivity was higher in waste pickers with consumption of rat meat (1/1: 100%) than that in those without this practice (3/89: 3.4%) (P = 0.04). In addition, the frequency of high (> 1.0 OD units) anti-*Leptospira* IgG antibody levels was higher in waste pickers with consumption of rat meat (1/1: 100%) than that in those without this practice (0/89: 3.4%) (P = 0.01). Other behavioral characteristics of waste pickers including contact with animals, drinking untreated water or unpasteurized milk, consumption of meat other than rabbit and rat meats, consumption of unwashed raw vegetables or fruits, contact with soil, and housing conditions showed P values > 0.10 by bivariate analysis. Multivariate analysis of the four variables (age, no education, and consumption of meat from rabbit and rat) with P values ≤ 0.10 obtained in the bivariate analysis showed that none of these variables was associated with *Leptospira* seropositivity.

## Discussion

The epidemiology of *Leptospira* infection in waste workers is largely unknown. To the best of our knowledge, there are no reports on the association of *Leptospira* infection with waste picking activity. Therefore, through this age- and gender-matched case-control study we aimed to determine such association in waste pickers in Durango City, Mexico. We found that prevalence of anti-*Leptospira* IgG antibodies was similar in waste pickers to that in controls from the general population. Therefore, our results suggest that waste picking is not associated with *Leptospira* exposure in Durango City. The lack of association between *Leptospira* exposure and waste picking is unexpected. The workplace (municipal solid waste transfer station) of waste pickers holds many animals including rats, mice and dogs feeding from the garbage. These animals are potential transmitters of *Leptospira*. Direct contact with urine of carrier animals may lead to *Leptospira* infection [15]. Many waste pickers do not use safety practices including wearing of hand gloves, and have skin injuries that might facilitate *Leptospira* infection. It is not clear why waste pickers had a low seroprevalence of *Leptospira* exposure. It is possible that animals living in or visiting the waste transfer station were not infected with *Leptospira*. It is also possible that waste pickers did not have skin abrasions or direct contact with urine of animals. The fact that most *Leptospira*-positive serum samples were weakly reactive suggests that exposure to *Leptospira* is not frequent among waste pickers. On the other hand, the finding of one strongly reactive sample draws the attention for the likelihood of acute infections among waste pickers. We searched for contributing factors of infection with *Leptospira* in the waste pickers. Bivariate analysis showed that *Leptospira* exposure was associated with increasing age, no education and consumption of rat meat. However, these associations were no longer found by multivariate analysis. This was most likely due to the small number of *Leptospira*-seropositive waste pickers found in the study. Nevertheless, the association of *Leptospira* exposure with consumption of rat meat is intriguing. In a search for this association in the medical literature, we did not find any report of such association. Rats are important reservoirs of *Leptospira* [16]. The waste picker who ate rat meat was the only case with high anti-*Leptospira* antibody levels. Infections with *Leptospira* by digestive route are considered exceptional, whereas skin abrasions are considered the main route of infection [3]. However, the finding of a strongly reactive serum sample from a waste picker with consumption of rat meat might suggest that this waste picker acquired *Leptospira* infection by the digestive route. We cannot rule out that *Leptospira* infection could be acquired through skin abrasions when manipulating rats before cooking. The waste picker with high anti-*Leptospira* IgG antibody levels referred to be suffering from bronchitis. *Leptospira* infection may lead to respiratory illness, and clinical manifestations vary from subtle clinical features to severe disease including pulmonary hemorrhage and respiratory distress syndrome [17-19]. We were unable to trace back further clinical data from the *Leptospira*-seropositive waste picker.

The present study has some limitations. The small sample size and a low number of *Leptospira*-seropositive waste pickers did not allow us to reach statistically significant results by multivariate analysis. In addition, the scanty clinical data obtained in the archival questionnaires did not allow us further determining an association between clinical features and *Leptospira* seropositivity.

### Conclusions

We demonstrate serological evidence of *Leptospira* exposure in waste pickers. In this first study about *Leptospira* exposure in waste pickers, results suggest that waste pickers are not at increasing risk for *Leptospira* exposure in Durango City, Mexico. Further research with a larger sample size to elucidate the association of *Leptospira* exposure with waste picking activity is needed.
